# Alkynyl‐Bipyridine–Based Conjugated Microporous Polymer Anode for Lithium Storage

**DOI:** 10.1002/smll.73965

**Published:** 2026-05-26

**Authors:** Yuanyuan Zhang, Yuanyuan Liu, Xiaorui Wang, Xinyi Zhao, Manhua Peng, Hongwei Fan, Lin Zhang

**Affiliations:** ^1^ Beijing Key Laboratory of Intelligent Design and Manufacturing for Hydrogen Energy Materials College of Chemical Engineering Beijing University of Chemical Technology Beijing P. R. China; ^2^ Key Laboratory of Power Station Energy Transfer Conversion and System Ministry of Education School of Energy Power and Mechanical Engineering North China Electric Power University Beijing P. R. China; ^3^ Institute For Solid State Physics Leibniz University Hannover Hannover Germany

**Keywords:** alkynyl linkages, bipyridine, conjugated microporous polymer, lithium‐ion storage, Sp‐ and sp2‐hybridized carbons

## Abstract

Conjugated microporous polymer (CMP) anode materials composed of sp‐ and sp^2^‐hybridized carbons are promising for lithium storage, owing to their *π*‐electron‐delocalization framework and abundant active sites. Unfortunately, such CMP‐based materials have rarely been reported, largely stemming from the synthetic challenge of achieving the predesigned structural characteristics (e.g., uniform pores). Herein, we synthesize an alkynyl‐bipyridine‐CMP (Alk‐Bpy‐CMP) which features a well‐defined six‐membered ring structure. The smallest structural unit consisting of alkynyl linkages, bipyridine, and aromatic rings processes high‐density distributed active sites and an extended *π*‐conjugated system. At a current density of 0.1 A g^−1^, the Alk‐Bpy‐CMP shows a specific capacity of 674 mAh g^−1^ after 100 cycles. This phenomenon can be ascribed to the preferential lithium storage behavior of bipyridine and alkynyl linkages, which possess a high proportion of storage sites. By boosting conductive property via reduced graphene oxide (rGO) encapsulation, the specific capacity was further enhanced to 1020 mAh g^−1^ after 200 cycles at 0.1 A g^−1^, approaching the theoretical specific capacity (1145.4 mAh g^−1^) and exceeding the majority of reported CMP. A stable 1000‐cycle discharge and charge test indicates the robust cycling performance of the resultant Alk‐Bpy‐CMP@rGO.

## Graphical abstract

In this work, a sp‐ and sp^2^‐hybridized alkynyl‐bipyridine conjugated microporous polymer (Alk‐Bpy‐CMP) was designed and synthesized. This structure possesses a regular hexagonal pore and abundant active sites, and the energy storage mechanism was revealed. Encapsulation with reduced graphene oxide (rGO) enhanced its electrical conductivity, and its specific capacity is higher than the majority of CMP as an anode for lithium‐ion batteries.

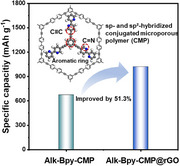

## 1 Introduction

Lithium‐ion batteries (LIBs) are being rapidly advanced for their high energy/volumetric density and power output in portable and automotive energy devices, while graphite, the mainstream anode material, is increasingly unable to meet the surging application demands [1, 2]. Enormous efforts have focused on developing diverse alternatives, notably low‐cost, eco‐friendly organic materials with multiple redox‐active centers [3, 4, 5, 6, 7]. Among them, conjugated microporous polymer (CMP) has attracted extensive attention due to their unique features such as insolubility in electrolytes and structural robustness [8, 9, 10, 11, 12]. Although some progress has been made in CMP materials, the majority of them are composed of sp^2^‐hybridized carbons, with C═O [13, 14] or C═N [15, 16], or aromatic rings [17] serving as the main redox‐active sites. These CMPs remain with inherent limitations of large charge transfer resistance, insufficient active sites, and limited capacity [18]. CMPs constructed with sp‐ and sp^2^‐hybridized carbons enable extended *π*‐conjugation via alkynyl linkages, while the sp‐hybridized moieties simultaneously act as extra active sites for ion storage, thereby boosting the specific capacity. Unfortunately, such materials have rarely been explored as anodes for LIBs, mainly due to the scarcity of multi‐redox‐center and multi‐electron redox‐active monomers, along with the challenges in fabricating well‐defined pore structures [19, 20, 21]. Most of them deliver a specific capacity of less than 800 mAh g^−1^, coupled with relatively poor cycling stability.

Herein, we design and synthesize an alkynyl‐bipyridine‐based CMP (Alk‐Bpy‐CMP) anode material for LIBs, where the alternating conjugation between sp‐hybridized alkynyl and bipyridine units with sp^2^‐hybridized carbons constructs a well‐defined six‐membered ring architecture. The C═N bonds, the alkynyl groups, and the aromatic rings can all serve as active sites for ion storage, demonstrating robust performance. The *π* bonds of alkynyl groups form an extensive conjugated system with sp^2^‐hybridized carbons in bipyridine and aromatic units, thus enhancing structural stability. Through encapsulation with reduced graphene oxide (rGO), the conjugated CMP establishes tight interfacial contact with rGO via strong *π‐π* stacking, enabled by their respective conjugated structures. This ensures efficient electron transfer, enhancing the CMP's conductivity as well as capacity, rate performance, and cycling stability. In situ characterization combined with theoretical simulations systematically reveals the lithium storage mechanism.

## 2 Results and Discussion

### 2.1 Preparation and Characterization of Alk‐Bpy‐CMP

**FIGURE 1:**
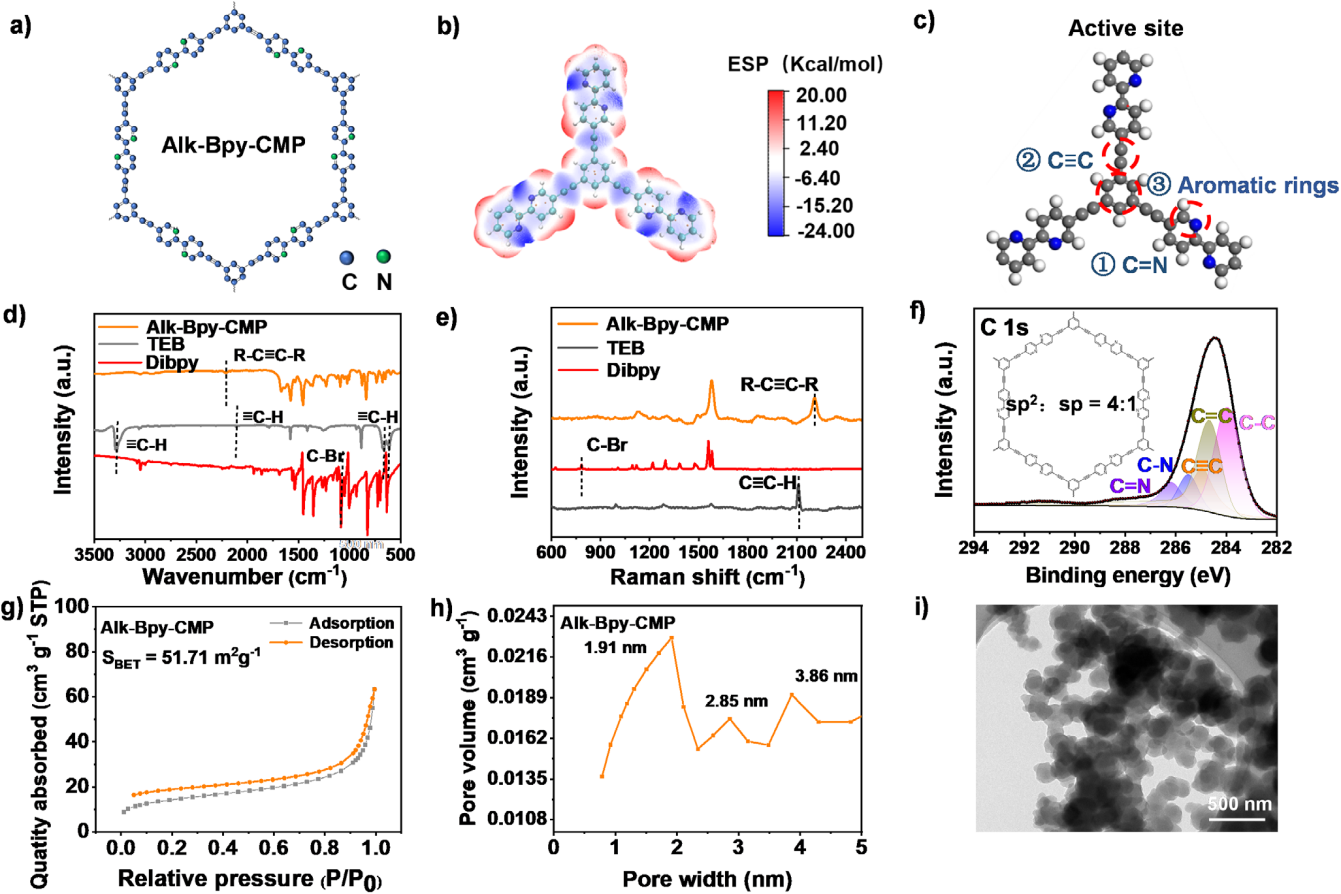
(a) Pre‐designed chemical structure of Alk‐Bpy‐CMP. (b) DFT calculations of the molecular electrostatic potential for Alk‐Bpy‐CMP. (c) Schematic of the active sites (The regions marked by red circles correspond to the active sites). (d) FT‐IR and (e) Raman spectra of the Alk‐Bpy‐CMP. (f) High‐resolution XPS spectra of deconvoluted C 1s spectra of Alk‐Bpy‐CMP. (g) Nitrogen adsorption and desorption isotherm and (h) the pore size distribution of Alk‐Bpy‐CMP. (i) TEM image of Alk‐Bpy‐CMP.

Figure 1a illustrates the well‐defined six‐membered ring structure of Alk‐Bpy‐CMP as designed. MESP was simulated to detect the redox‐active sites (red represents the positive and blue represents the negative) though Density Functional Theory (DFT) (Figure 1b). It can be seen that the promising main active sites for energy storage are near the pyridine nitrogen, the alkynyl groups, and aromatic rings. Besides, the active sites of the two monomers were also predicted: the active sites of TEB are alkynyl bonds and aromatic rings, whereas those of Dibpy are C═N bonds and aromatic rings, which is consistent with the active sites of Alk‐Bpy‐CMP. This further confirms the location of the active sites in Alk‐Bpy‐CMP (Figure S1). Moreover, the site adjacent to the C═N units displays the most negative electrostatic potential. It can be reasonably inferred that the C═N groups serve as the primary ion‐storage sites, followed by the alkynyl groups and then the aromatic rings (Figure 1c). Figure S2 confirms that Alk‐Por‐CMP exhibits a stable range from 1.00 × 10^−3^ S cm^−1^ to 1.25 × 10^−3^ S cm^−1^. Moreover, as shown in Figure S3, we compared Alk‐Bpy‐CMP with other sp^2^‐hybridized conjugated polymers. The results reveal a much smaller charge‐transfer resistance, confirming that Alk‐Bpy‐CMP possesses fast charge‐transfer kinetics and excellent electron‐transport capability.

Alk‐Bpy‐CMP was synthesized via the Sonogashira‐Hagihara cross‐coupling reaction of 1,3,5‐triethynylbenzene (TEB) and 5,5'‐dibromo‐2,2'‐bipyridine (Dibpy) (Figure S4) [22]. As shown in the FT‐IR spectra (Figure 1d), Alk‐Bpy‐CMP reveals a new characteristic peak at ∼2200 cm^−1^ belonging to the vibration of C≡C bonds [23, 24], and the C─Br peak at ∼1070 cm^−1^ [25]. The ≡C─H peaks at ∼3260, 2110, 670, and 610 cm^−1^, originating from Dibpy and TEB, respectively, are no longer observed, confirming the successful condensation of ≡C─H and C─Br units [26, 27]. The Raman spectrum of the as‐synthesized Alk‐Bpy‐CMP exhibits a new characteristic peak at ∼2210 cm^−1^ ascribed to C≡C stretching vibrations (Figure 1e) [28]. The C─Br bonds (∼715 cm^−1^) of Dibpy and ≡C─H bonds (∼2130 cm^−1^) of TEB disappear, suggesting the complete polymerization between them. Additionally, the Raman spectrum exhibits only a prominent G peak with a negligible D peak, further demonstrating that the reaction successfully constructed an ordered conjugated system. High‐resolution XPS spectra of deconvoluted C 1s spectrum at ∼286.4, 286.0, 285.5, 284.8, and 284.4 eV correspond to C═N, C─N, C≡C (C sp), C═C (C sp^2^), and C─C (C sp^3^), respectively (Figure 1f) [29, 30]. Notably, the calculated area ratio of sp^2^ to sp is close to 4:1 consistent with the pre‐designed ring structure (inset in Figure 1f) with a hexagonal pore of Alk‐Bpy‐CMP. If the reaction only formed linear chain structures without cyclization, characteristic signals of unreacted ≡C─H and C─Br groups would remain in the FT‐IR and Raman spectra, which is inconsistent with our experimental results. This further confirms that the product successfully constructs a well‐defined six‐membered ring skeleton. The N 1s spectrum displays two characteristic peaks at ∼399.5 and 398.5 eV, corresponding to C─N and C═N bonds, respectively (Figure S5a) [31, 32]. In addition, nitrogen adsorption and desorption isotherm (Figure 1g,h) reveals a Brunauer‐Emmett‐Teller (BET) surface area of 51.7 m^2^ g^−1^ and a pore width distribution ranging from 1.91 to 3.86 nm for Alk‐Bpy‐CMP. SEM image (Figure S5b) reveals that Alk‐Bpy‐CMP is composed of nanoparticles with a diameter of about 100 nm. TEM (Figure 1i) confirms that these nanoparticles are uniform in size, and evidence of a layered morphology can be discerned. The water contact angle value of 93.3° (Figure S5c) indicates a characteristic hydrophobic framework of Alk‐Bpy‐CMP. As shown in Figure S6, the contact angles of Alk‐Bpy‐CMP, TEB, and Dibpy with the electrolyte were measured to be 24.5°, 20.9°, and 28.4°, respectively. All three materials show much smaller electrolyte contact angles than 90°, indicating excellent wettability. This superior affinity promotes full electrolyte penetration into the electrode pores, optimizes interfacial contact, reduces charge‐transfer resistance, and accelerates Li^+^ migration kinetics. The XRD shows an amorphous structure of Alk‐Bpy‐CMP (Figure S7). The amorphous state indicates that Alk‐Bpy‐CMP possesses six‐membered ring structures with disordered stacking characteristics. Consequently, it should be clarified that there is no contradiction between the well‐defined molecular framework and the amorphous characteristic revealed by XRD. Furthermore, TGA curve (Figure S8) demonstrates that the mass loss of Alk‐Bpy‐CMP below 200°C is mainly due to residual volatile solvents and moisture. Nearly 70% of the initial mass remains at 800°C verifies its outstanding thermal stability.

### 2.2 Electrochemical Performances of Alk‐Bpy‐CMP

**FIGURE 2:**
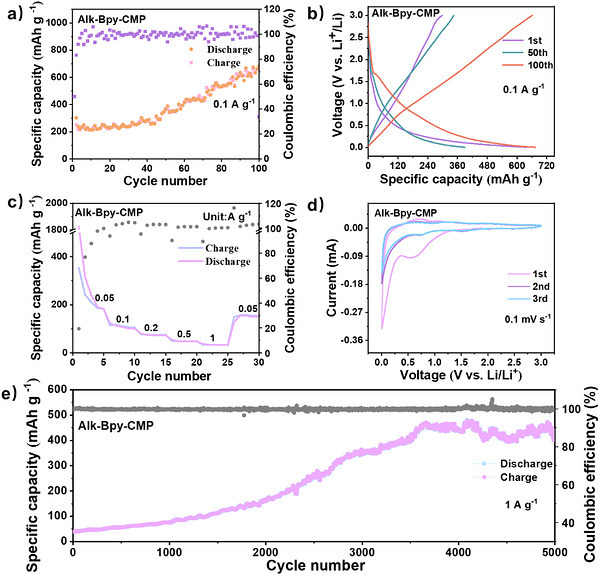
(a) Cycling performances of the Alk‐Bpy‐CMP electrode at the current density of 0.1 A g^−1^. (b) GCD curves of the Alk‐Bpy‐CMP electrode at the first, the 50th, and the 100th cycle, respectively. (c) Rate performance of the Alk‐Bpy‐CMP electrode at different current densities. (d) CV curve of Alk‐Bpy‐CMP electrode at a scan rate of 0.1 mV s ^−1^. (e) Long‐term cycling test of Alk‐Bpy‐CMP at 1 A g^−1^.

Alk‐Bpy‐CMP was employed as an anode material for LIBs, and its electrochemical performance was evaluated. Figure 2a shows the cycling performance of the Alk‐Bpy‐CMP at 0.1 A g^−1^. An increase in capacity after 40 cycles is observed, deriving from the gradual activation of the material and the sufficient exposure of redox sites [33, 34, 35]. After 40 cycles, repeated Li^+^ intercalation and deintercalation progressively exposes the Alk‐Bpy‐CMP electrode fully to the electrolyte and activates initially inaccessible active sites, which is a typical characteristic of organic electrode materials [36, 37]. Figure 2b presents Galvanostatic charge‐discharge (GCD) curves of Alk‐Bpy‐CMP electrode at the current density of 0.1 A g^−1^ for the first, 50th, and 100th cycles. It is worth noting that the Alk‐Bpy‐CMP electrode exhibits discharge/charge capacities of 637/298 mAh g^−1^ with coulombic efficiency (CE) of 46.7% in the first cycle. The low initial CE primarily originates from electrolyte decomposition and the solid electrolyte interphase (SEI) layer formation. This phenomenon could be improved by pre‐lithiation strategy and encapsulating with highly conductive materials [38, 39]. Following activation, the Alk‐Bpy‐CMP electrode delivers discharge/charge capacities of 389/345 mAh g^−1^ with CE of 88.6% at the 50th cycle, which further increase to 674/661 mAh g^−1^ with 98% CE at the 100th cycle. This performance is significantly higher than that of the TEB and Dibpy control electrodes (Figure S9a,c). The rate performance of the as‐prepared polymer electrodes was investigated (Figure 2c). In the first few tens of cycles, the material is not fully activated, and its active sites are not sufficiently exposed. Therefore, compared with Figure 2a, the capacity exhibits a decreasing trend at the current density of 0.1 A g^−1^. After cycling at various rates, the capacity can be back restored to 200 mAh g^−1^ at 0.05 A g^−1^, demonstrating the good rate capability of Alk‐Bpy‐CMP electrode, which is also better than TEB and Dibpy electrodes (Figure S9b,d). CV curve (Figure 2d) reveals an irreversible reduction peak at 0.5‐1.0 V during the first cycle, which can be further traced to the decomposition of electrolyte and the formation of SEI. SEM images of the Alk‐Bpy‐CMP electrode before and after 100 cycles verify the excellent structural stability of Alk‐Bpy‐CMP during repeated cycling (Figure S10). The superior electrochemical properties of the Alk‐Bpy‐CMP electrode originates from its sp‐ and sp^2^‐hybridized carbons framework and hexagonal pore structure. Long‐term cycling tests of Alk‐Bpy‐CMP were carried out at a high current density of 1 A g^−1^(Figure 2e). The results show that the specific capacity of the material increases gradually with cycling, and stabilizes at around 500 mAh g^−1^ after 3500 cycles. This high capacity is well maintained up to 5000 cycles, fully demonstrating its excellent structural stability and cycling durability.

### 2.3 Lithium Storage Mechanism of Alk‐Bpy‐CMP

**FIGURE 3:**
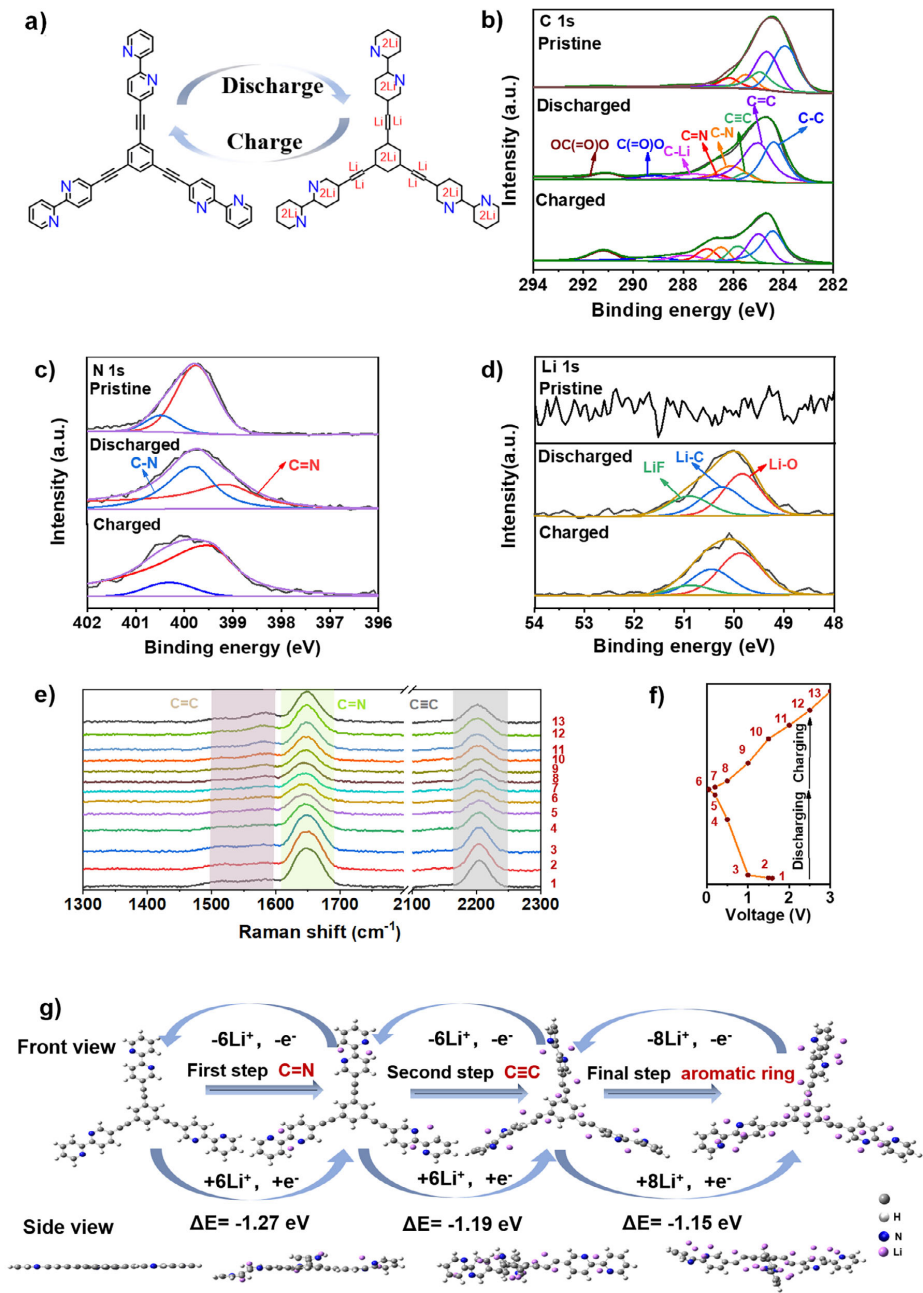
(a) Illustration of the Li storage mechanism of Alk‐Bpy‐CMP electrode. High‐resolution XPS spectra of deconvoluted (b) C 1s and (c) N 1s spectra of Alk‐Bpy‐CMP electrode at different states. (d) High‐resolution XPS spectra of deconvoluted Li 1s spectra of Alk‐Bpy‐CMP electrode at different states. Alk‐Bpy‐CMP electrode at different voltages during the charging and discharging process: (e) Raman spectra, (f) discharging‐charging points, (g) Schematic showing lithium storage sequence of the Alk‐Bpy‐CMP active sites.

The lithium storage mechanism of Alk‐Bpy‐CMP was systematically explored via characterization and computational simulations. First, XPS measurements were conducted to confirm the reaction mechanism during discharging and charging process (Figure 3a). Figure 3b shows that the high‐resolution deconvoluted C 1s XPS spectra peaks at ∼286.5, 286.0, 285.6, 284.8, and 284.4 eV can be attributed to C═N, C─N, C≡C (C sp), C═C (C sp^2^), and C─C (C sp^3^), respectively. The intensities of the C═N, C≡C, and C═C peaks decrease after discharging, while the C─N and C─C peaks intensify. All these functional groups can be recovered when recharged to 3.0 V. Remarkably, the decrease of the C═C unit is relatively slight, as lithiation of the C≡C bonds (C≡C to C═C) compensates for the reduction of aromatic rings. Evidently, assuming all C≡C units undergo a four‐electron storage reaction to form C─C units, the peak area of C─C and C═C groups would be inconsistent with the XPS spectrum in Figure 3b (otherwise the peak area of C─C groups will be comparatively large). This result further supports the two‐electron redox mechanism for each C≡C unit (from C≡C to C═C rather than C─C units) in the Alk‐Bpy‐CMP electrode due to the strong steric‐hindrance and inductive effect of Li‐C═C─Li against more lithium‐ion storage. The reversible change of peaks at ∼399.7 (C─N) and 399.0 eV (C═N) in N 1s spectra also proof the reversible redox reaction of C═N units of bipyridine (Figure 3c). Additional peaks observed at ∼287.5, 289.0, and 291.0 eV, corresponding to C─Li, C(═O)O, and OC(═O)O units arising from SEI formation, are irreversible after recharging. This confirms that organic compounds (such as ROCO_2_Li, R═CH_2_/CH_2_CH_3_) are formed via decomposition of the electrolyte and the formation of SEI during the first‐cycle lithiation process. Additionally, Li 1s spectrum can be deconvolved into Li─F, Li─C, and Li─O peaks in the discharged state, and cannot fully recovered after recharging (Figure 3d), which can be assigned to the formation of Li‐F, Li_2_CO_3_, and ROCOOLi from the electrolyte and SEI evolution [40, 41].

In situ Raman spectra of the Alk‐Bpy‐CMP electrode under different voltages are shown to further clarify the lithiation/delithiation mechanism (Figure 3e,f). First, the peak intensity of C≡C units (at ∼2205 cm^−1^) decreases, indicating that the C≡C bonds participate in Li^+^ storage. Conversely, the peak intensity of C≡C recovers in the subsequent recharging process, suggesting reversible redox behavior with Li^+^. Second, the peak ascribed to C═N units at ∼1647 cm^−1^ decreases significantly during discharge and is fully restored in the subsequent charging process, revealing reversible Li^+^ storage at the C═N units in the Alk‐Bpy‐CMP electrode. Finally, the characteristic peaks of aromatic rings (at ∼1580 and 1515 cm^−1^) gradually diminish during the discharging process. This phenomenon arises from the electrostatic interaction between Li^+^ cations and the *π*‐electron system of the aromatic rings. Notably, the weakened peaks at ∼1580 and 1515 cm^−1^ during discharging correspond to sp^2^‐hybridized carbons in the aromatic rings. The incomplete disappearance of the peaks at ∼1580 and 1515 cm^−1^ after discharge also indicate that the sp^2^ carbons generated by C≡C lithiation and the subsequent formation of C═C─Li moieties partially compensate for the depletion of aromatic sp^2^ carbons during the process. Owing to the reversible redox reaction, all peaks regain their sharp and narrow profiles during the subsequent charging process, with the peaks associated with aromatic rings fully restored. This phenomenon provides direct evidence for the reversible Li^+^ storage at the aromatic rings in the Alk‐Bpy‐CMP electrode. All curves in Figure 3e correspond one‐to‐one to the scatter points in Figure 3f from bottom to top, with identical serial numbers matched between the two graphs.

DFT was implemented to further investigate the reversible lithium‐ion storage sequence of Alk‐Bpy‐CMP, especially for the alkynyl groups and bipyridine units. First, the calculated HOMO and LUMO energy levels of Alk‐Bpy‐CMP are −6.12 and −2.30 eV, respectively (Figure S11). The higher value of HOMO than TEB (−6.79 eV) and Dibpy (−6.69 eV) monomers indicates Alk‐Bpy‐CMP with sp‐ and sp^2^‐hybridized carbons can excite more electrons, and have stronger reduction ability, resulting in higher specific capacity. The lower LUMO energy of Alk‐Bpy‐CMP relative to those of the TEB (−1.57 eV) and Dibpy (−2.08 eV) monomers also indicates its stronger electron affinity, which facilitates electron acceptance during redox processes. Compared to TEB (ΔE = 5.22 eV) and Dibpy (ΔE = 4.61 eV) monomers, the lowest HOMO‐LUMO gap (ΔE = 3.82 eV) of Alk‐Bpy‐CMP is beneficial to the electron transfer, which further indicates the superior intrinsic electronic conductivity of Alk‐Bpy‐CMP. All original calculated data for the simulated HOMO and LUMO orbitals are listed in Table S1. Sequentially, lithium ions were then introduced at different active sites of Alk‐Bpy‐CMP to unravel the ion storage sequence among the active sites at different positions (Figure 3g). The negative adsorption energy implies the spontaneous reaction between Alk‐Bpy‐CMP and lithium ions. The site adjacent to C═N units exhibit the most negative state and can accommodate 6Li ions (ΔE = −1.27 eV) for each smallest structural unit, indicating its strongest affinity for cations (such as Li^+^) owing to its higher electronegativity of N than C atoms. Then, the active sites of C≡C bonds show a moderate negative value across the entire structure. Each C≡C bond could stably accept 2Li ions by optimization with an optimized adsorption energy of ΔE = –1.19 eV. Finally, near aromatic rings also display a negative value and could incorporate 8Li ions in each smallest structural unit with ΔE = –1.15 eV. Theoretical calculations and geometric optimizations reveal the efficient utilization of redox‐active sites proximal to pyridine nitrogen atoms, C≡C bonds, and aromatic rings within the Alk‐Bpy‐CMP framework.

### 2.4 Preparation and Electrochemical Performances of Alk‐Bpy‐CMP@rGO

**FIGURE 4:**
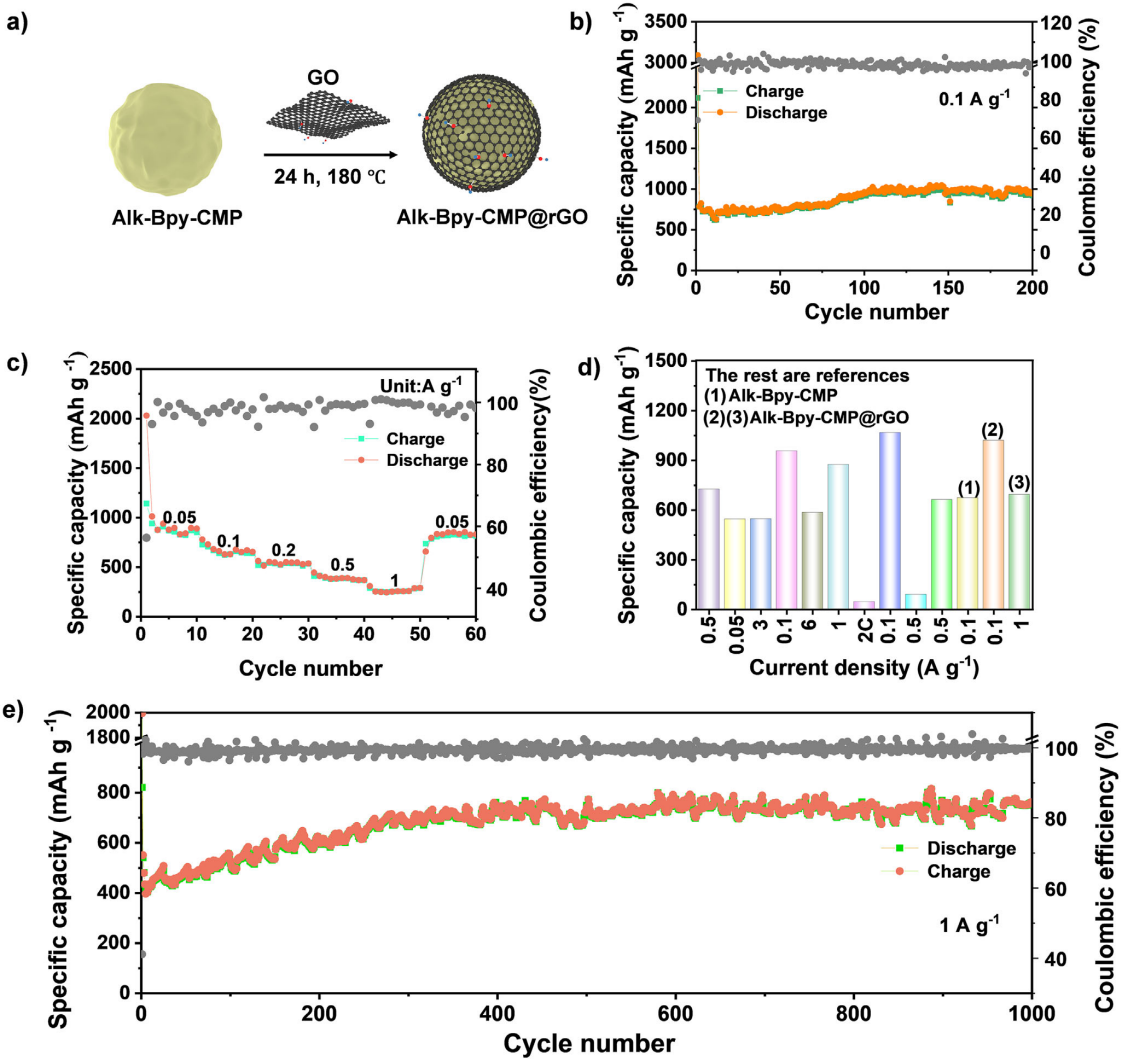
(a) Illustration of preparing Alk‐Bpy‐CMP@rGO via rGO encapsulation on Alk‐Bpy‐CMP. (b) Cycling performances of the Alk‐Bpy‐CMP@rGO electrode at the current density of 0.1 A g^−1^. (c) Rate capability of the Alk‐Bpy‐CMP@rGO electrode at different current densities. (d) Comparison of the electrochemical performance with other reported CMPs anodes for LIBs (please see details in Table S3). (e) Long‐term cycle test of the Alk‐Bpy‐CMP@rGO electrode at the current density of 1 A g^−1^.

Electrochemical performances of the Alk‐Bpy‐CMP were further improved by encapsulation of rGO to enhance the conductive property (Figure 4a) [42]. The conjugated planes of CMP and rGO form an intimate interfacial integration via *π‐π* stacking interactions [43]. In this case, electron transport relies not only on the conjugated system of Alk‐Bpy‐CMP but also proceeds rapidly through the rGO network, leading to a significant reduction in charge transfer resistance [44]. Energy‐dispersive X‐ray spectroscopy (EDS) was conducted on GO, rGO, Alk‐Bpy‐CMP, and Alk‐Bpy‐CMP@rGO, with the corresponding results shown in Figures S13–S16. The elemental mass fractions of the above materials are listed in Table S2. Alk‐Bpy‐CMP exhibits a relatively small oxygen content of 12.82 wt.%, which is mainly attributed to the slight oxidation of alkynyl groups. In comparison, the oxygen content in Alk‐Bpy‐CMP@rGO increases to 21.74 wt.%. As can be seen from Table S2, oxygen elements still remain even after the conversion of GO to rGO. Therefore, we can reasonably infer that the increased oxygen content in Alk‐Bpy‐CMP@rGO originates from rGO. Meanwhile, the uniformly distributed oxygen signals observed in Figure S16 further verify that rGO is homogeneously encapsulated around Alk‐Bpy‐CMP. Figures S17 and S18 indicate that the interaction between Alk Bpy CMP and rGO is dominated by physical contact, without obvious formation of new covalent bonds or significant structural damage to the polymer skeleton. They are tightly combined through these physical interactions, mainly including *π‐π* stacking, van der Waals forces, and electrostatic interactions. These interfacial interactions construct a continuous high‐speed electron transport pathway, reduce interfacial contact resistance, extend the electron delocalization range, and promote rapid interfacial charge transfer. The ultralow impedance observed in the electrochemical impedance spectroscopy (EIS) of rGO confirms its superior electrical conductivity (Figure S20a), demonstrating that material encapsulation represents an effective strategy for enhancing the electrical conductivity of the material. In addition, we further measured the electrical conductivity of the Alk‐Bpy‐CMP@rGO composite. As shown in Figure S20b, the electrical conductivity of Alk‐Bpy‐CMP@rGO remains stable at 0.20 S cm^−^^1^, which is three orders of magnitude higher than that of Alk‐Bpy‐CMP (Figure S2). At current density of 0.1 A g^−1^, the Alk‐Bpy‐CMP@rGO electrode obtains the discharge capacities of 1020 mAh g^−1^ for 200 cycles (Figure 4b), which is higher than those previously reported for other CMPs (Figure 4d; Table S3). After encapsulating each of the two monomers with rGO individually, their electrochemical performance was indeed enhanced, yet still inferior to that of Alk‐Bpy‐CMP (Figure S21). Figure S22a illustrates that at a current density of 0.1 A g^−1^, the Alk‐Bpy‐CMP@rGO electrode exhibits initial charge/discharge capacities of 1305.75/1820.96 mAh g^−1^ and an initial CE of 71.7% in the initial cycle, a value significantly higher than the 46.7% CE of the pristine Alk‐Bpy‐CMP electrode. While the initial charge/discharge capacities are only 801.57/1990.36 mAh g^−1^ (CE of 40.2%, Figure S22b) and 705.55/1283.78 (CE of 54.9%, Figure S22c) for TEB@rGO and Dibpy@rGO electrodes, respectively. Figure 4c and Figure S22 reveal the specific capacities are 1012.83, 779.75, 563.52, 446.2, 310.21, and 920.21 mAh g^−1^ at current densities of 0.05, 0.1, 0.2, 0.5, 1, and 0.05 A g^−1^, respectively, demonstrating excellent capacity retention and rate performance relative to the TEB@rGO and Dibpy@rGO electrodes (Figure S23). A long‐term 1000‐cycle charge‐discharge test at a high current density of 1 A g^−1^ yields capacities of 693.9/696.1 mAh g^−1^, indicating the outstanding cycling durability (Figure 4e). Compared with Alk‐Bpy‐CMP, the CV curve of Alk‐Bpy‐CMP@rGO presents a remarkably enlarged peak area, further verifying the improved specific capacity after rGO encapsulation (Figure S24). Meanwhile, the Alk‐Bpy‐CMP@rGO electrode exhibits a higher peak current, manifesting the improved reaction activity. The peak area of CV curves of the two monomers also increases, which is consistent with the electrochemical performance results (Figure S25). Cycling and rate tests of the rGO anode demonstrate that rGO makes a certain contribution to the capacity of Alk‐Bpy‐CMP@rGO, but the majority of the capacity still originates from Alk‐Bpy‐CMP (Figure S26). As can be seen from Figure S26b, the capacity of rGO remains at 250 mAh g^−1^ after 100 cycles, while the capacity of Alk‐Bpy‐CMP@rGO reaches 1000 mAh g^−1^ at the same stage. The EIS spectrum of the Alk‐Bpy‐CMP@rGO electrode displays a smaller semicircle diameter than that of the pristine Alk‐Bpy‐CMP electrode (Figure S27), confirming the enhanced electrical conductivity by rGO encapsulation and thus leading to superior reaction kinetics [45, 46]. Simultaneously, the EIS results of the two monomers encapsulated with rGO align well with the observations for Alk‐Bpy‐CMP@rGO (Figure S28). The CV curves recorded at various scan rates from 0.2 to 1.0 mV s^−1^ (Figure S29a) and the corresponding linear plot of peak current versus scan rate collectively demonstrate the capacitive‐controlled charge storage mechanism of the Alk‐Bpy‐CMP@rGO electrode (Figure S29b). The b values of the redox peaks in Alk‐Bpy‐CMP@rGO are 0.643 and 0.798, also representing capacitive‐controlled redox kinetics. We have successfully assembled full cells by pairing the Alk‐Bpy‐CMP@rGO anode with a commercial NCM811 cathode. The schematic diagram of the battery working mechanism is shown in Figure S30a. The full cells were evaluated at a large current density of 0.2 A g^−1^, and delivered a reversible specific capacity of approximately 100 mAh g^−1^ even after 100 cycles, demonstrating good application potential of the material in practical lithium‐ion full cells (Figure S30b).

## 3 Conclusion

An alkynyl‐bipyridine‐based conjugated microporous polymer with both sp and sp^2^ hybridization carbons was synthesized, which displays a well‐defined ring structure. The C═N bonds within the bipyridine units, alkynyl bonds, and aromatic rings can all act as active sites for lithium‐ion storage. Since the adsorption energy near C═N is lower than that of alkynyl bonds, the ion storage priority is C═N, succeeded by alkynyl bond and aromatic ring. Following encapsulation with rGO, the electrical conductivity of Alk‐Bpy‐CMP shows an approximately 4.4‐fold enhancement, leading to accelerated reaction kinetics. The resultant Alk‐Bpy‐CMP@rGO exhibits a specific capacity of 1020 mAh g^−1^ after 200 cycles at 0.1 A g^−1^ and 693.9 mAh g^−1^ after 1000 cycles at 1 A g^−1^, which is competitive with most state‐of‐the‐art polymer anode materials. This work would provide guidance for the design of sp‐ and sp^2^‐hybridized carbon‐based CMPs with high specific capacity and long‐term cycling durability for electrochemical energy storage.

## Conflicts of Interest

The authors declare no competing financial interest.

## Supporting information




**Supporting File**: smll73965‐sup‐0001‐SuppMat.docx.

## Data Availability

The data that support the findings of this study are available from the corresponding author upon reasonable request.
