# Supplementary material for: Alkynyl‐Bipyridine–Based Conjugated Microporous Polymer Anode for Lithium Storage
Source: Small. 2026 May 26;22(40):e73965. doi: 10.1002/smll.73965 (PMC13378614; doi:10.1002/smll.73965)
Supplement: Supplementary file 1 — Supporting File: smll73965‐sup‐0001‐SuppMat.docx. [file SMLL-22-e73965-s001.docx]

Supporting Information

Alkynyl-Bipyridine–Based Conjugated Microporous Polymer Anode for Lithium Storage

Yuanyuan Zhang,^a^ Yuanyuan Liu,^a^ Xiaorui Wang,^a^ Xinyi Zhao,^a^ Manhua Peng,^b,*^ Hongwei Fan,^a,*^ and Lin Zhang ^c,*^

a Beijing Key Laboratory of Intelligent Design and Manufacturing for Hydrogen Energy Materials, College of Chemical Engineering, Beijing University of Chemical Technology, Beijing 100029, P. R. China

b Key Laboratory of Power Station Energy Transfer Conversion and System, Ministry of Education, School of Energy Power and Mechanical Engineering, North China Electric Power University, Beijing 102206, P. R. China

c Institute for Solid State Physics, Leibniz University Hannover, Appelstrasse 2, Hannover 30167, Germany

Corresponding Author.

a,* E-mail: fanhongwei@mail.buct.edu.cn (Hongwei Fan)

b,* E-mail: pmh502@ncepu.edu.cn (Manhua Peng)

c,* E-mail: l.zhang@fkp.uni-hannover.de (Lin Zhang)

**Experimental section**

**Materials:** 1,3,5-Triethynylbenzene (TEB, >95.0%), 5,5'-Dibromo-2,2'-bipyridyl (Dibpy >98.0%), and copper (Ⅰ) iodate (CuI, ≥99.5%) were purchased from Aladdin. Tetrakis (triphenylphosphine) palladium (Pd(PPh_3_)_4_, ≥99.8%) was purchased from Macklin, N, N dimethylformamide (DMF, ≥99.5%), triethylamine (Et_3_N, ≥99.5%), methanol (MeOH, ≥99.5%), ethyl alcohol (EtOH, ≥99.7%), dichloromethane (CH_2_Cl_2_, ≥99.5%), and N-methyl-2-pyrrolidone (NMP, ≥99.0%) were provided by Sinopharm. Lithium metal (>99%) used as the anode was obtained from Beijing Research Institute of Nonferrous Metals, China. The electrolyte, 1 M LiPF_6_ in EC/DEC (1:1, Vol%), was of analytical purity and purchased from Duoduo Chemical Reagent. Polyvinylidene difluoride (PVDF) of excellent grade purity was supplied by Shenzhen BAK Battery Company. All materials were used as received without further purification.

**Synthesis of Alk-Bpy-CMP：** Under Ar atmosphere, anhydrous DMF (5.0 mL) and Et_3_N (1.5 mL) were added into a 100 mL heat-resistant glass bottle containing TEB (15 mg, 0.1 mmol), Dibpy (47.1 mg, 0.15 mmol), Pd(PPh_3_)_4_ (34.6 mg) and CuI (5.0 mg) and sealed. The mixture was stirred at 100 °C in an oil bath for 72 h. After cooling down to room temperature, the yellow precipitate was collected via filtration and washed with copious MeOH and CH_2_Cl_2_. To exchange residual solvent and by-products from the pores to obtain purified products, the precipitate was extracted by Soxhlet extraction in CH_2_Cl_2_ for 24 h. Then, the resultant Alk-Bpy-CMP was collected and dried in the oven at 60 °C. Finally, the yellow powder (Alk-Bpy-CMP, 30 mg, yield rate: 84.1%) could be obtained.

**Synthesis of Alk-Bpy-CMP@rGO：** Firstly, 0.5 g graphene oxide (GO) was uniformly dispersed in 250 mL deionized water, and a 2 mg mL^-1^ GO dispersion was obtained through ultrasound and prolonged stirring. Subsequently, the prepared Alk-Bpy-CMP (30 mg) was dispersed in the aforementioned GO dispersion (10 mL), sonicated and stirred for another 1 h. Subsequently, the mixture was transferred to a 100 mL Teflon-lined vessel, sealed tightly, and heated at 180 °C for 24 h. The resulting black gel was dried in vacuum environment at 60 °C overnight to obtain the black powder product of Alk-Bpy-CMP@rGO.

**Materials characterization：** The morphologies of the products were characterized using a scanning electron microscope (SEM, ZEISS Sigma 360) equipped with an energy-dispersive X-ray spectrometer (EDX), as well as a transmission electron microscope (TEM, JEOL JEM-F200). The elemental analysis (EA) was performed using an the Elementary Vario MICRO cube. The crystallographic structure and phase composition of the samples were characterized by Power X-ray diffraction (PXRD, Rigaku-2038) with Cu Kα radiation at λ = 1.5406 Å. Fourier transform infrared (FT-IR, Thermo Fisher Scientific Nicolet iS20) and Raman spectroscopy (Horiba LabRAM HR Evolution) were employed to record the corresponding spectra, aiming to analyze the chemical bonds and functional groups in the samples. N₂ physisorption measurements were performed at 77 K on a Micromeritics ASAP 2460 system following pretreatment (samples were degassed at 150 °C for 12 h). X-ray photoelectron spectroscopy (XPS, Thermo Scientific K-Alpha) was employed to characterize the surface chemical compositions of all composites, using Al Kα radiation as the excitation sources. Thermogravimetric analysis (TGA, HITACHI STA200) was carried out at a heating rate of 10 °C min^-1^ to assess the structural stability of the samples, while electrode kinetics were analyzed using a CHI760D electrochemical workstation.

**Electrochemical tests：** To prepare the working electrode, the active material (Alk-Bpy-CMP) was thoroughly mixed with Super P and polyvinylidene fluoride (PVDF) binder at a mass ratio of 7:2:1. NMP was added during mixing to form a homogeneous slurry, which was then coated onto copper foil (thickness: 90 μm, diameter: 12 mm) and dried in vacuo at 80 °C for 10 h to gain the final working electrode. The mass loading of the active material was ~ 0.7-1.5 mg per copper foil. However, the mass of each electrode disk is approximately 1 mg. The working electrode was paired with Li metal and assembled into CR2032 coin cells in an argon-filled glove box (Vigor, SG1200/750TS; H_2_O < 0.1 ppm, O_2_ < 0.1 ppm) for electrochemical measurements. The electrolyte was 1.0 M LiPF_6_ dissolved in a 1:1 (v/v) mixture of ethylene carbonate (EC) and diethyl carbonate (DEC). The porous polypropylene (Celgard 2500) was selected as the separator. Galvanostatic charge–discharge (GCD) tests and long-term cycling performance were conducted on a NEWARE battery test system over a voltage window of 0.005-3.0 V. Specific capacities were calculated based on the total mass of the active material. Electrochemical impedance spectroscopy (EIS, frequency range: 0.01–100000 Hz) and cyclic voltammetry (CV, scan rate: 0.1-1.0 mV s^-1^) were performed on a CHI760D electrochemical workstation within the same voltage window of 0.005-3.0 V.

For full-cell assembly, the anode was prepared as described above, while NCM811 was employed as the cathode instead of lithium metal. The NCM811 cathode was fabricated by mixing the active material, Super P conductive carbon, and PVDF binder at a mass ratio of 7:2:1 in NMP to form a homogeneous slurry. The slurry was then coated onto Al foil and vacuum-dried at 80 °C for 10 h, followed by punching into 12 mm-diameter disks. Prior to full-cell assembly, the Alk-Bpy-CMP@rGO anode was electrochemically prelithiated in a half-cell configuration. The electrolyte consisted of 1 M LiPF_6_ in EC/DEC (v/v = 1:1), and a Celgard 2500 membrane was used as the separator. During prelithiation, the half-cells were first cycled once at 0.05 A g^-1^ within a voltage window of 0.005-3.0 V, and then fully discharged to 0.005 V at 0.1 A g^-1^ to form a lithium-embedded CMP structure.The full cells were tested in the voltage range of 2.5-4.2 V, and all electrochemical measurements were performed at 30 °C.

**Theoretical simulations and calculation：** The structures of the monomers and their complexes were fully optimized using the B3LYP method with the 6-311G(d) basis set. Vibrational frequency calculations were performed to confirm that the optimized structures contained no imaginary frequencies. The intermolecular interaction energy ($\text{∆E}_{\text{int}}$)was calculated by

$\text{ΔE}_{\text{int}}\text{=}{\text{（}\text{E}}_{\text{nA/B}}\text{-n}\text{E}_{\text{A}}\text{-}\text{E}_{\text{B}}）/n$ (1)

where E_A_ and E_B_ are the energies of the isolated molecule and E_nA/B_ is the total energies of the complex geometry, respectively; n is the number of Li ions in the complex. All the calculations were realized by Gaussian 16 software.^[1]^ The HOMO/LUMO were calculated at same level. The molecular electrostatic potentials (MESP) of the isolated monomers were represented via the Multiwfn 3.7 program.^[2]^

**Figure S1.** DFT calculations of the molecular electrostatic potential of a) TEB and b) Dibpy.

**Figure S2.** Electrical conductivity of Alk-Bpy-CMP.

Fig. S3. Comparison of impedance between Alk-Bpy-CMP and other sp2-hybridized carbons CMPs and polymers.

**Figure S4.** Schematic diagram of the synthesis and molecule structure of Alk-Bpy-CMP.

The Alk-Bpy-CMP product was successfully prepared a facile one-step strategy: 1,3,5-Triethynylbenzene (TEB), 5,5'-Dibromo-2,2'-bipyridyl (Dibpy), and a mixed solvent were added in Ar atmosphere and then sealed for reaction.^[9]^

**Figure S5.** a) N 1s XPS spectra of Alk-Bpy-CMP. b) SEM image of Alk-Bpy-CMP c) Water droplet profile with corresponding contact angle value on the surface of pressed Alk-Bpy-CMP powders.


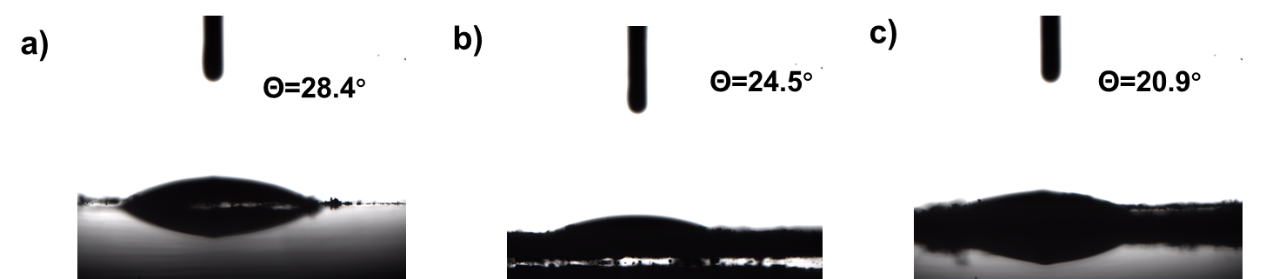


**Figure S6.** a), b), and c) represent the contact angles of Alk-Bpy-CMP, TEB, and Dibpy with the electrolyte, respectively.

**Figure S7.** XRD of Alk-Bpy-CMP.

**Figure S8.** TGA curve of Alk-Bpy-CMP with the heating rate of 10 ℃ min^-1^.

**Figure S9.** Cycling performances of the a) TEB and c) Dibpy electrode at the current density of 0.1 A g^-1^. The rate capability of the b) TEB and d) Dibpy electrode at different current densities.

**Figure S10.** SEM images of the Alk-Bpy-CMP a) before and b) after 100 cycles.


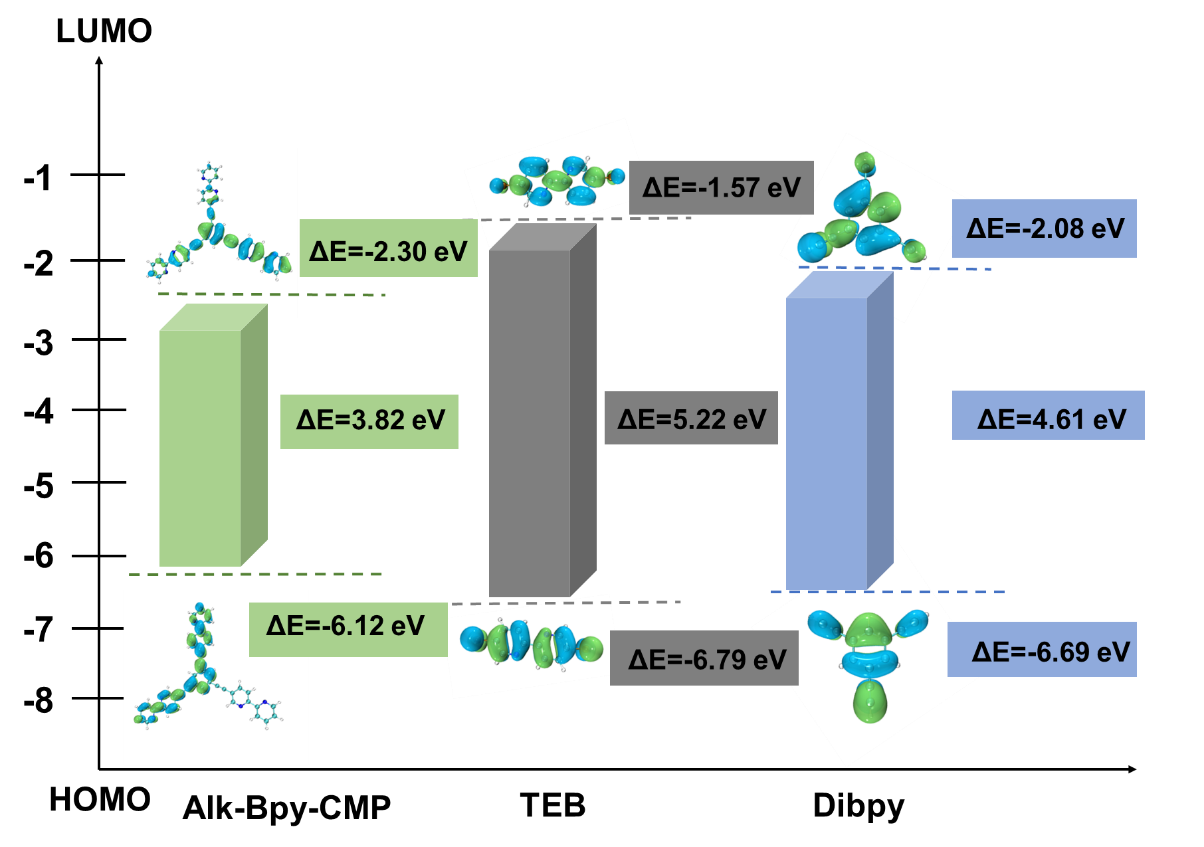


**Figure S11.** The simulated HOMO and LUMO energy levels of Alk-Bpy-CMP, TEB and Dibpy.

**Figure S12.** a) SEM, b) TEM and c) HR-TEM images of Alk-Bpy-CMP@rGO.

Alk-Bpy-CMP particles are evenly encapsulated in the lamellar structure of reduced graphene oxide. TEM images of Alk-Bpy-CMP@rGO proved the highly crystalline graphene layers with lattice stripes uniformly separate Alk-Bpy-CMP, leading to an eﬀective improvement in the overall conductivity of Alk-Bpy-CMP.


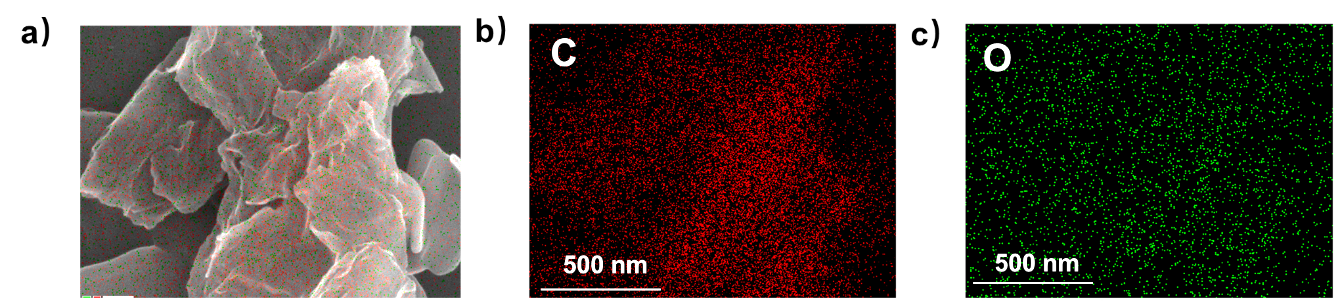


**Fig. R13.** Elemental mapping of GO.


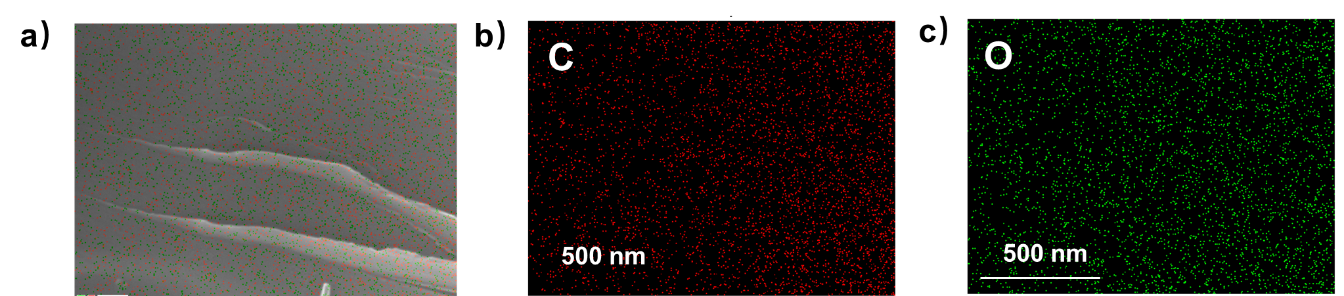


**Figure S14.** Elemental mapping of rGO.


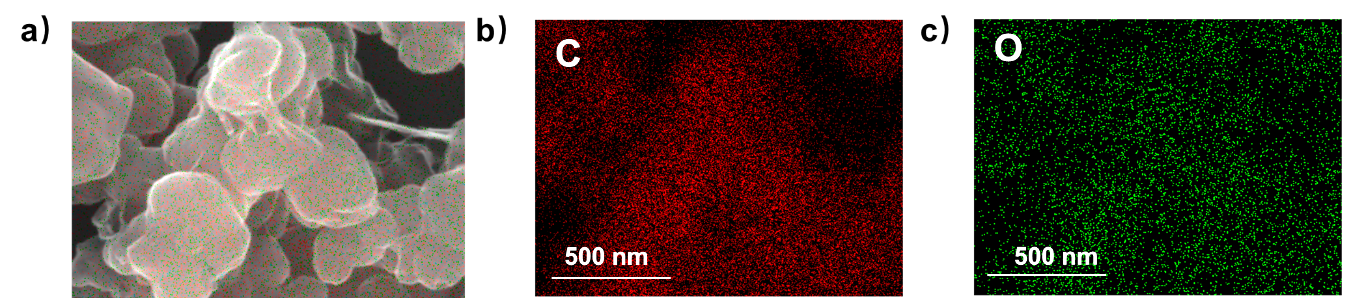


**Figure S15.** Elemental mapping of Alk-Bpy-CMP.


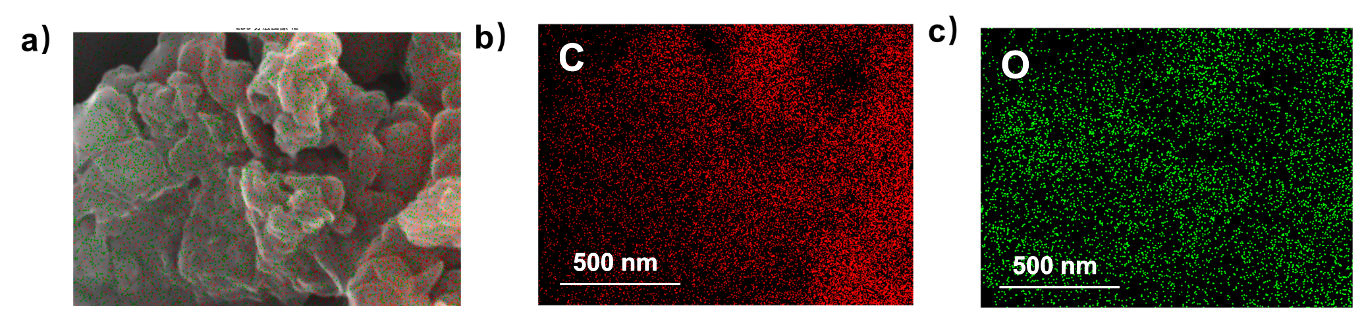


**Figure S16.** Elemental mapping of Alk Bpy CMP@rGO.

**Figure S17.** FT-IR spectra of monomer Alk-Bpy-CMP@rGO, rGO and the Alk-Bpy-CMP.

The spectra of Alk-Bpy-CMP@rGO closely resemble the Alk-Bpy-CMP, indicating that the introduction of rGO does not damage the abundant active groups in Alk-Bpy-CMP, which can still serve as eﬀective coordination sites for cation storage.

**Figure S18.** Raman spectra of monomer Alk-Bpy-CMP@rGO, rGO and the Alk-Bpy-CMP.

Incorporation of rGO significantly intensifies both the D-band (at 1340 cm^-1^, corresponding to structural defects or amorphous carbon) and G-band (at 1580 cm^-1^, corresponding to crystalline graphite) in the Raman spectrum. More importantly, the characteristic peak of C≡C bonds (typically located at ~ 2100–2200 cm^-1^) derived from Alk-Bpy-CMP is well preserved with no obvious peak shift. This phenomenon not only confirms the successful loading of rGO onto the Alk-Bpy-CMP framework but also indicates that the intrinsic conjugated structure of Alk-Bpy-CMP remains intact after encapsulation.^[10]^

**Figure S19.** XRD of monomer Alk-Bpy-CMP@rGO, rGO and the Alk-Bpy-CMP.

The Alk-Bpy-CMP@rGO only exhibits a broad peak near 26°, corresponding to the (002) plane. This once again successfully demonstrates the successful combination of rGO with Alk-Bpy-CMP.^[11]^


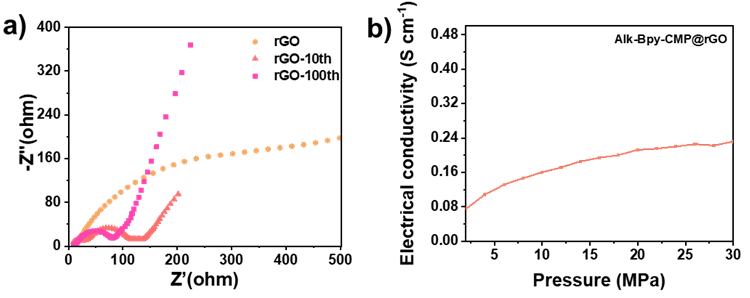


**Figure S20.** a) Nyquist plots of rGO electrode at different cycles. b) Electrical conductivity of Alk-Bpy-CMP@rGO.

**Figure S21.** Cycling performances of the a) TEB@rGO and b) Dibpy@rGO electrode at the current density of 0.1 A g^-1^.

**Figure S22.** The discharge-charge curves of the a) Alk-Bpy-CMP@rGO, b) TEB@rGO, and c) Dibpy@rGO electrode at different cycle.

**Figure S23.** The rate capability of the a) TEB@rGO and b) Dibpy@rGO electrode at different current densities.

**Figure S24.** Cyclic voltammetry curve of Alk-Bpy-CMP@rGO at a scan rate of 0.1 mV s ^-1^.

**Figure S25.** Cyclic voltammetry curve of a) TEB, b) Dibpy, c) TEB@rGO, and d) Dibpy@rGO at a scan rate of 0.1 mV s ^-1^.

**Figure S26.** a) The first three cycles of cyclic voltammograms of the rGO. b) Cycling performances of the rGO electrode at the current density of 0.1 A g^-1^. c) The rate capability of the rGO electrode at different current densities.

**Figure S27.** Nyquist plots of a) Alk-Bpy-CMP b) Alk-Bpy-CMP@rGO at different cycles.

**Figure S28.** Nyquist plots of a) TEB, b) Dibpy, c) TEB@rGO, and d) Dibpy@rGO electrode at different cycles.

**Figure S29.** a) Cyclic voltammograms profiles at different scan rates of the Alk-Bpy-CMP@rGO electrode. b) Log (i) versus log (v) plots of the Alk-Bpy-CMP@rGO electrode.

Based on the relationship between peak current (i) and scanning rate (v): i = av^b^, the b values can be calculated to quantitatively measure the contribution of capacitive-control to the electrochemical process of the electrode.


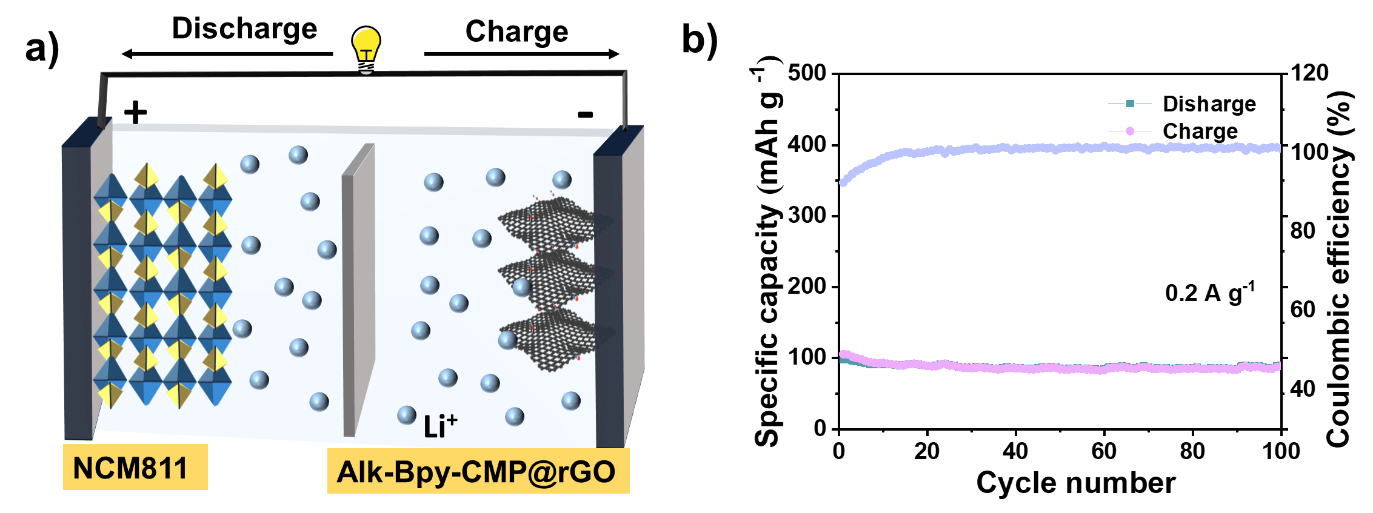


**Figure S30.** a) Schematic diagram of the full cell. b) Cycling performance at 0.2 A g^-1^ for the Alk-Bpy-CMP@rGO//NCM811 full cell.

**Table S1** Raw data table for theoretical calculations

|  | Alk-Bpy-CMP | TEB | Dibpy |
| --- | --- | --- | --- |
| HOMO | -6.119585 eV | -6.789675 eV | -6.689288 eV |
| LUMO | -2.296886 eV | -1.573205 eV | -2.078719 eV |
| ΔE | 3.82 eV | 5.22 eV | 4.61 eV |

**Table S2** Elemental mass table

|  | C (wt%) | N (wt%) | O (wt%) |
| --- | --- | --- | --- |
| GO | 84.95 | — | 15.05 |
| rGO | 89.65 | — | 10.35 |
| Alk-Bpy-CMP | 68.79 | 18.39 | 12.82 |
| Alk-Bpy-CMP@rGO | 70.84 | 7.42 | 21.74 |

**Table S3** Comparison of the performance of other CMPs in the anode of lithium-ion batteries in the literature.

| Abbreviation  for Compounds | Capacity/  Cycles (mA h g^-1^) | Current Density (mA g^-1^) | Voltage  Range (V) | Ref. |
| --- | --- | --- | --- | --- |
| Azo-fused CMP^1^ | 725/500 | 500 | 0.005-3.0 | [12] |
| Co-PCMPs^2^ | 438/100 | 1000 | 0.01-3.0 | [13] |
| CMP-Por^3^ | 546/5000 | 3000 | 0.01-3.0 | [14] |
| CNT@TAPT-BTPA^4^ | 957.5/50 | 100 | 0.01-3.0 | [15] |
| TCPP^5^ | 585.47/3000 | 6000 | 0.01-3.0 | [16] |
| PHATN-CMP^6^ | 874.3/1000 | 1000 | 0.05-3.0 | [17] |
| IEP-11-E12^7^ | 46.7/9000 | 298 | 0.001-3.0 | [18] |
| Alkynyl-CPF^8^ | 1068/700 | 100 | 0.005-2.5 | [19] |
| PT^9^ | 90/1000 | 500 | 0.005-3.0 | [20] |
| P3DDT^10^ | 663/1000 | 500 | 0.005-3.0 | [20] |
| Alk-Bpy-CMP@rGO | 1020/200 | 100 | 0.005-3.0 | This work |
| Alk-Bpy-CMP@rGO | 693/1000 | 1000 | 0.005-3.0 | This work |
| Alk-Bpy-CMP | 674/100 | 100 | 0.005-3.0 | This work |

1: Polymer of AzoBT comprising azo and benzothiadiazole units; 2: Porphyrin-based CMPs; 3: Porphyrin-based CMPs; 4: Two imine-linked triazine-based CMPs with carbon nanotubes (CNT); 5: π-conjugated tetrakis (4-carboxyphenyl) porphyri; 6: Hexaazatrinnphthalene (HATN)-based CMPs; 7: Anthraquinone-based CMPs; 8: Alkynyl-linked covalent phenanthroline framework; 9-10: Thiophene-Containing CMPs.

**References**

[1] S. Grimme. **2006**, 27, 1787-1799.

[2] T. Lu, F Chen, J. Comput. Chem. **2012**, 33, 580-592.

[3] Xie, M. G., Li, C. G., Ren, S. Y., Ma, Y., Chen, X. B., Fan, X. F., Han, Y., Shi, Z., and Feng, S. H., J. Mater. Chem. A 2022, 10, 15089-15100.

[4] Weng, T. H., Mohamed, M. G., Sharma, S. U., Chaganti, S., Samy, M. M., Lee, J. T., and Kuo, S. W., ACS Appl. Energy Mater. 2022, 5, 14239-14249.

[5] Xiang, S., Wang, S. Q., Zhang, G. B., Irshad, M. S., Ma, Z. J., and Li, M., J. Power Sources 2024, 602.

[6] Li, H., Wu, J., Li, H. B., Xu, Y. L., Zheng, J., Shi, Q. F., Kang, H. W., Zhao, S. Q., Zhang, L. H., Wang, R., Xin, S., Zhou, T. F., and Zhang, C. F., Chem. Eng. J. 2022, 430.

[7] Ba, Z. H., Wang, Z. X., Luo, M., Li, H. B., Li, Y. Z., Huang, T., Dong, J., Zhang, Q. H., and Zhao, X., ACS Appl. Mater. Interfaces 2020, 12 (1).

[8] Shi, R. J., Liu, L. J., Lu, Y., Wang, C. C., Li, Y. X., Li, L., Yan, Z. H., and Chen, J., Nat. Commun. 2020, 11 (1).

[9] X. Zhuang, F. Zhang, D. Wu, N. Forler, H. Liang, M. Wagner, D. Gehrig, M. R. Hansen, F. Laquai, X. Feng, Angew. Chem. Int. Ed. **2013**, 52, 9968-9672.

[10] W. Li, H. Xu, H. Zhang, F. Wei, L. Huang, S. Ke, J. Fu, C. Jing, J. Cheng, S. Liu, Nat. Commun. **2023**, 14, 5235.

[11] M. Liu, D. Jiang, Y. Fu, G. Zheng Chen, S. Bi, X. Ding, J. He, B. H. Han, Q. Xu, G. Zeng, Angew. Chem. Int. Ed. **2024**, 63, E202317015.

[12] W. Ma, C. Zhang, X. Gao, C. Shu, C. Yan, F. Wang, Y. Chen, J. H. Zeng, J.-X. Jiang, J. Power Sources. **2020**, 453, 227868.

[13] L. Shu, J. Yu, Y. Cui, Y. Ma, Y. Li, B. Gao, H.-g. Wang, Int. J. Hydrog. Energy. **2022**, 47, 10902-10910.

[14] Y. Yang, J. Yuan, S. Huang, Z. Chen, C. Lu, C. Yang, G. Zhai, J. Zhu, X. Zhuang, J. Power Sources. **2022**, 531, 231340.

[15] L. Lian, K. Li, L. Ren, D. Han, X. Lv, H.-g. Wang, Colloids Surf. A: Physicochem. Eng. Asp. **2023**, 657, 130496.

[16] H. Wu, J. Zhang, X. Du, M. Zhang, J. Yang, J. Zhang, T. Luo, H. Liu, H. Xu, G. Cui, Chem. Commun. **2019**, 55, 11370-11373.

[17] S. Li, Y. Wang, X. Zhang, X. Lv, H.-g. Wang, Mater. Today Commun. **2024**, 38, 107768.

[18] A. Molina, N. Patil, E. Ventosa, M. Liras, J. Palma, R. Marcilla, Adv. Funct. Mater. **2020**, 30, 1908074.

[19] Y. Cao, H. Fang, C. Guo, W. Sun, Y. Xu, Y. Wu, Y. Wang, Angew. Chem. Int. Ed. **2023**, 62, E202302143.

[20] C. Zhang, Y. He, P. Mu, X. Wang, Q. He, Y. Chen, J. Zeng, F. Wang, Y. Xu, J. X. Jiang, Adv. Funct. Mater. **2018**, 28, 1705432.
